# Microclimate drives intraspecific thermal specialization: conservation perspectives in freshwater habitats

**DOI:** 10.1093/conphys/coab006

**Published:** 2021-04-12

**Authors:** Fabrizio Bartolini, Folco Giomi

**Affiliations:** 1 NEMO Nature and Environment Management Operators S.R.L., Viale Mazzini 26, 50132 Florence, Italy; 2 Via Maniciati 6, 35129 Padua, Italy

**Keywords:** Freshwater snail, microclimatic niche, relict species, respiration, thermal adaptation, thermoregulatory behaviour

## Abstract

Endemic and relict species are often confined to ecological refugia or over fragmented distributions, representing priority conservation subjects. Within these sites, the individual population may realize distinct niches to a varying degree of specialization. An emblematic example is provided by freshwater species segregated in thermal-mineral springs, where individuals may face highly diverse microclimates in limited geographic areas. Downscaling the characterization of physiological traits to microclimatic niches becomes pivotal to adopt effective conservation measures in these heterogeneous habitats. *Melanopsis etrusca* (Brot, 1862) is an endangered relict snail endemic to a small number of thermal-mineral streams in central Italy. Here we describe the thermal tolerance of two populations of *M. etrusca* inhabiting streams with distinctly different thermal regimes, investigating the extent of physiological and behavioural specialization to such diverse microclimatic niches. The comparison of oxygen consumption rates of a population dwelling in temperate streams, characterized by seasonal temperature fluctuations (12–27°C), with a population experiencing constantly hot water (35–38°C) revealed the absence of any seasonal or geographic effect on metabolic compensation. Conversely, mobility performances were maximized in the population inhabiting the hot stream. Interestingly, here, the snails exhibited emersion behaviour outside the water, triggered by temperatures above 37°C. In the field, individuals of this population are observed inactive on stream banks, conceivably to minimize the metabolic cost that otherwise would be induced by remaining in the hot water. Only a few individuals from the temperate stream exhibited the same behaviour when exposed to elevated temperatures, suggesting the exaptation of a pre-existing trait during the evolutionary process of adaptation to hot waters. The present results provide elements for the best practice in future programmes aimed at reintroducing stocks of threatened species across heterogeneous habitats. Our study further underlines the relevance of downscaling data collection for endangered species conservation in order to recognize microclimatic specializations.

## Teaser

Downscaling the study of the endangered relict snail, *Melanopsis etrusca*, at the level of populations, inhabiting geographically separated thermal springs in central Italy, we identified intraspecific mechanisms of local specialization. We advocate that the fit of physiological and behavioural traits to different microclimatic niches should be included to design effective conservation actions.

## Introduction

Temperature and its variability across space and time are leading physical factors shaping the complexity and distribution of life (Angilletta, 2009). Virtually, all species are characterized by a thermal niche (i.e. the temperature range within which fitness is maintained), the breadth of which is directly influenced by abrupt, daily and seasonal temperature fluctuations (Tracy and Christian, 1986; Huey, 1991). Ectothermic organisms, in particular, are strictly subjected to thermal regimes (i.e. the temporal and spatial variation of environmental temperatures), which influences, to a different extent, all biological processes, such as growth efficiency, reproductive output, cost of maintenance or mobility and metabolic performances (Angilletta *et al*., 2002; Angilletta, 2009; Huey, 1991; Pörtner, 2010). On an evolutionary time scale, natural selection shapes the breadth of thermal niches, promoting the differentiation of thermal specialist vs. generalist species as a function of the higher or lower variation of the thermal regime (Gilchrist, 1995; Verberk *et al*., 2016). In this regard, the understanding of the thermal requirements of different species becomes pivotal in order to accurately define their microclimatic niche and consequently to make reliable predictions of their vulnerability to environmental changes and to adopt precise conservation measures (Huey, 1991).

Recently, the importance of downscaling climatic data collection to match the environmental variation actually experienced by species has been proposed (Potter *et al*., 2013; Varner and Dearing, 2014; Pincebourde *et al*., 2016). We believe that this guidance should be paralleled by downscaling the characterization of physiological processes to the level of individual microclimatic niches. This approach often remains overlooked, despite its potential to both clarify the natural history of distinct populations and drive effective conservation strategies.

Spring habitats, both cold and hot, often represent ecological refuges from past environmental changes, such as those associated to the quaternary climate cycles. Thus, these relict or extreme ecosystems constitute optimal models to test adaptive responses of aquatic organisms to local or harsh environments (Specziár, 2004). At an evolutionary scale, the segregation of populations within spring habitats, imposed for example by the onset of adverse environmental conditions, may determine the diversification of phenotypes and life traits (Brown and Feldmeth, 1971; Mladenka and Minshall, 2001), ultimately causing speciation (Hershler *et al*., 2007). Examples include endemic fish species of the genus *Eremycthis* and *Cyprinodon* from north American deserts (Vinyard, 1996; Lema and Nevitt, 2006) and the springsnails of the genus *Pyrgulopsis* and *Trochidrobia* from desert springs in North America and Australia, respectively (Liu and Hershler, 2012; Murphy *et al*., 2012). The extremely localized distribution range of endemic species dwelling in spring habitats poses severe conservation challenges, both due to the intrinsic vulnerability of the populations to natural stochastic extinction events and to human impacts locally endangering spring ecosystems.

The freshwater snails of the genus *Melanopsis* were widespread in the Mediterranean region and in central Europe during the warm and humid phases of the middle Miocene and Pliocene (e.g. Esu, 1980; Esu and Girotti, 1989; Geary, 1990; Guerra-Merchán *et al*., 2010; Neubauer *et al*., 2015). The onset of the Pleistocene glacial cycles determined the extinction of melanopsid species from most parts of their northern geographic range (e.g. Esu *et al*., 1993; Di Bella *et al*., 2002). Three relict species are currently associated to headwaters of streams fed by thermo-mineral springs in Romania (*Melanopsis parreyssii*; IUCN category ‘CR’, Fehér, 2011; Sîrbu and Benedek, 2016, however more recently assessed it as extinct), northern Spain (*Melanopsis penchinati*; Pujante *et al*., 1988; IUCN category ‘CR’, Martínez-Ortí, 2011) and central Italy (*Melanopsis etrusca*; Cianfanelli *et al*., 1991; IUCN category ‘EN’, Cianfanelli, 2010).

The poor conservation status of *M. etrusca* is the result of several anthropogenic pressures, which have historically determined its decline, locally causing population extinctions (Cianfanelli *et al*., 1991).

Populations of *M. etrusca* are currently known to occur in five thermal streams in southern Tuscany. For geological reasons, the springs that feed these streams differ from each other in terms of their physical features and chemical composition (Bartolini *et al*., 2017). In particular, water thermal regimes range from temperate, with seasonal fluctuations of water temperature (12–27°C), to constantly hot (35–38°C). The streams inhabited by *M. etrusca* are therefore, due to their sizable differences in thermal regimes, an ideal case study to apply an ecophysiological comparison downscaled to the microclimatic level. In parallel, the understanding of the degree of specialization attained by separate populations in response to local conditions is of extreme importance to develop effective measures for the conservation of this threatened species.

*Melanopsis etrsuca* belongs to the Caenogatsropoda (Colgan *et al.*, 2007), a group composed by operculate and gill-breathing snails. In general, a more limited tolerance to desiccation is observed in gill breather compared to pulmonate snails, which are able to use atmospheric oxygen for respiration (Poznańska *et al.*, 2015; Koopman *et al.*, 2016). However, caenogastropods are usually have thick shell and an operculum, allowing species dwelling near-shore habitat subjected to water level fluctuation to avoid desiccation through aestivation (Poznańska *et al.*, 2015). Aestivation is the behaviour of withdrawal into the shell, which associates with aerobic hypometabolism (adaptive, as compared to metabolic depression, which implies either an adaptive or a capacity limited response; e.g. Marshall and McQuaid, 2020). Under severe environmental conditions, snails can even switch to anaerobic facultative metabolism while aestivating (e.g. Storey and Storey, 2012; Ferreira *et al.*, 2003). Such a strategy is particularly well developed among species adapted to periodically fluctuating environments, like temporary ponds or streams. Like other freshwater gill-breathing snails, *M. etrusca* occurs in running waters, inhabiting streams nourished by permanent springs, which prevents significant water level fluctuation.

The aim of our study was to downscale the characterization of the thermal physiology of *M. etrusca* to population level, focusing on two streams with different thermal conditions. Specifically, we compared the physiological thermal sensitivity and the behavioural response to heat between a population living in a temperate stream, characterized by a thermal regime that fluctuates on a seasonal basis, and a population confined to a constantly hot stream. Implications for the conservation of the species are discussed in light of the experimental evidence.

## Materials and methods

### *Melanopsis etrusca* distribution and habitat

**Figure 1:**
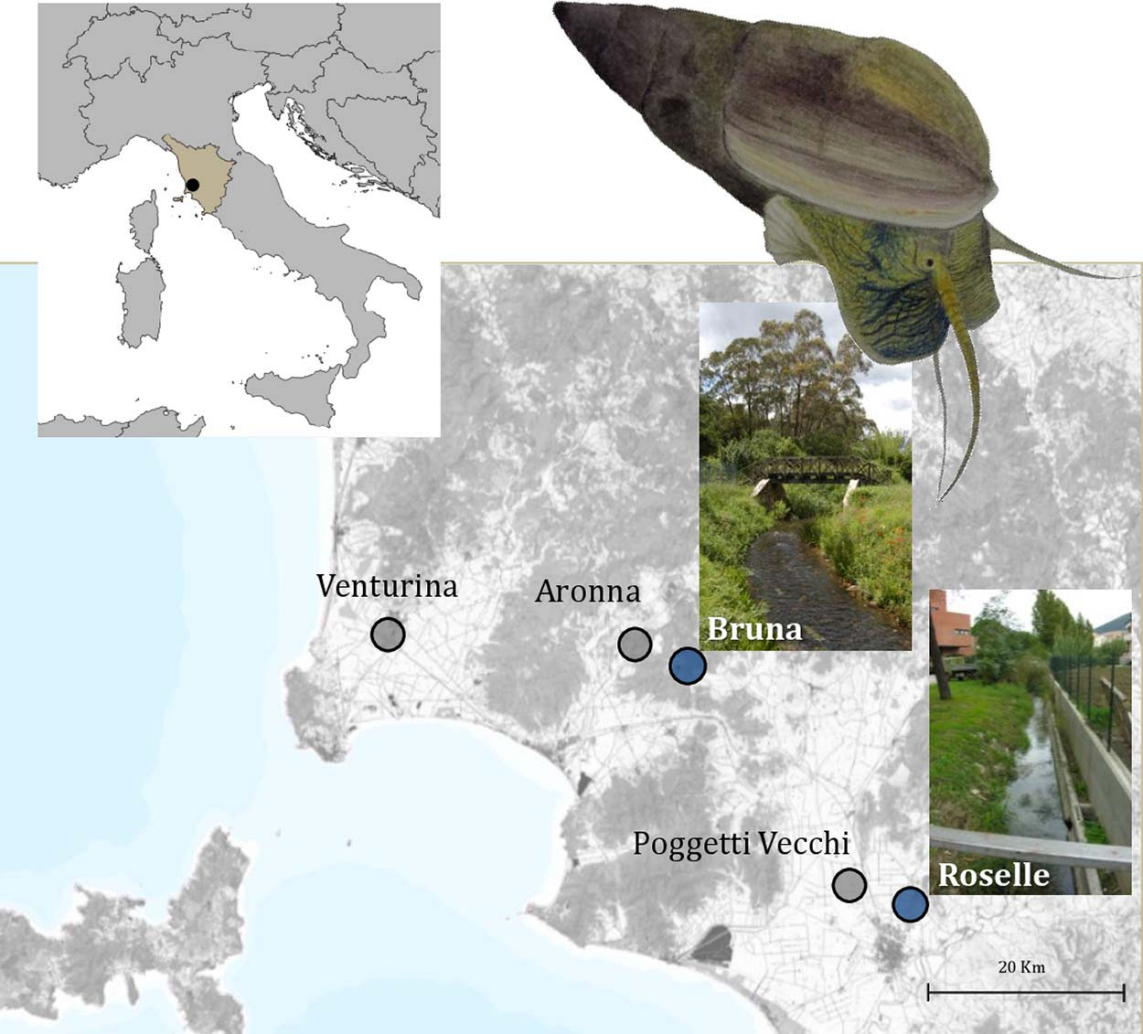
Current distribution of extant *M. etrusca* populations, with photographic details of the two study sites, Bruna and Roselle. An image of a live snail from the Bruna population is also shown (original watercolor by F. Bartolini).

The freshwater gastropod *M. etrusca* (Brot, 1862) (Gastropoda; Mollusca) is found in five locations in southern Tuscany (Venturina, Aronna, Bruna, Poggetti Vecchi and Roselle; Fig. 1), inhabiting the upper course of streams characterized by thermo-mineral waters (Bencini *et al*., 1977; Celati *et al*., 1991). One of these populations, Poggetti Vecchi, is severely threatened by anthropogenic stream desiccation, running the risk of local extinction, as has occurred to six other populations in recent years (Neiber *et al*., 2020). The thermal regimes of the streams inhabited by *M. etrusca* are highly heterogeneous (Bartolini *et al*., 2017). Specifically, streams are characterized by water temperatures ranging between 30 and 35°C in Venturina, 19–23°C in Aronna, and a higher water temperature (33°C, Bartolini pers. observ.) in Poggetti Vecchi, but the annual temperature variation in these locations has not yet been recorded. The stream in Roselle has the highest water temperature among all locations and a constant thermal regime throughout the year (35–38°C) (Bartolini and Giomi, in prep). The stream in Bruna is an effluent of Accesa Lake, which is fed by a system of cooler springs from the lake bed. This stream is characterized by broad seasonal temperature variation that spans from 12°C in winter (February) to 27°C in summer (August; Bartolini and Giomi in prep).

To investigate the degree of specialization of *M. etrusca* in these heterogeneous freshwater habitats, we measured the physiological (oxygen consumption rate and mobility performance) and the behavioural responses to a range of experimental thermal conditions, focusing on snails from the two most diverse streams: the constantly hot stream (Roselle) and the seasonally variable stream (Bruna).

### Experimental protocol

Animal sampling and the subsequent experiments on the Bruna population were carried out on two cohorts of snails (shell length, 2.5–16.6 mm) collected twice during the year, to encompass the seasonal variation of the thermal niche from 20°C in spring (2010) to 27°C in summer (2011). Due to the steady nature and narrow breadth of the thermal niche experienced by the population in Roselle, sampling was only conducted once at this site in spring when water temperatures of 36–38°C were recorded (2010).

We transported live snails from the stream to the laboratory in polystyrene boxes, in order to limit thermal stress; the maximum thermal excursion during transportation was less than 2°C over 1 hour. Subsequently, snails were housed at the same temperature measured in the stream at the moment of their collection. Water temperature inside 15 l aquaria was adjusted by means of heating cords connected to a precision thermostat and the systems were thermally isolated by polystyrene panels mounted on the lateral and bottom walls of the aquaria. This setup allowed fine-scale water temperature regulation and compensation with a precision of ±0.2°C. Snails were provided with continuously aerated water and natural stones, collected in their respective streams to reflect the chemical features of the individual sites and minimize the effect of sampling. All animals were acclimated for 2 days to laboratory conditions prior to experimentation, which also allowed for their recovery from translocation and handling stress.

The experimental protocol for all analytical set-ups involved stepwise procedures, either decreasing or increasing temperature, at an average rate of 2°C^*^h^−1^. Specifically, starting from each particular control temperature, snails were cooled or heated to the lowest or highest value setting the assay temperatures along the temperature ramp every 3–4°C for the physiological trials and every 1°C for the behavioural observations. A similar rate of temperature variation for the two experiments has low ecological relevance for the species, since it inhabits environments particularly stable on a short time scale. *M. etrusca* experiences sporadic variation in its thermal environment only when individuals emerge on the stream banks or crawl in very shallow waters. However, we adopted this stepwise rate of temperature variation in order to meet the criteria for reliable comparison with similar case studies (Giomi *et al*., 2016; Leung *et al*., 2019). We also chose to test the spring cohort of Bruna between 8 and 32°C, covering a very large thermal window, in order to explore the physiological response at low temperatures. The summer cohort of Bruna was tested considering the upper part of the thermal tolerance window, between 28 and 36°C. Finally, the population from Roselle was tested between 25 and 40°C.

Due to the endangered conservation status of this species, shell length was assumed as a proxy of body mass (e.g. Chaparro *et al*., 2019; McClain *et al*., 2006) to avoid killing of experimental animals. At the end of the experiments, snails were returned to their original streams.

### Oxygen consumption rate and mobility performance

Respirometric measurements were carried out using the electrode micro-respiration system MRS-8 (Unisense, Aarhus, Denmark), provided with eight, individually-stirred, micro-respiration glass chambers (approximate volume 4 ml). Respirometric chambers were filled with filtered (0.2 μm) and sterilized stream water to reduce the confounding effect of respiration by micro-organisms.

When the assay temperature was reached, the glass chambers, each housing an individual snail (shell length, 3.5–14.1 mm), were sealed and oxygen-concentration was measured. A second measurement was performed after 15 min and the oxygen-consumption rate was extrapolated from the difference between the two measurements. Values of oxygen consumption rate were standardized for snail shell length. For each assay temperature, different groups of 24 specimens were tested.

Mobility performance was tested by snail righting trials performed at each assay temperature. Snails (shell length, 2.6–15.8 mm) were placed individually in small plastic containers with the shell opening facing upwards. After a short period of immobility (less than 30 seconds), probably due to the disturbance caused by handling, this position stimulated an instinctive reaction of the snails to return to the natural crawling position. The time spent for snails to right themselves was recorded and considered as a proxy of snail mobility performance. For each experimental temperature, we tested separate groups of 30 specimens.

### Behavioural response

Since many individuals of the Roselle population were observed aestivating a few centimetres above the water level on the stream banks, a behavioural experiment was designed to test if water temperature is the driving factor for this migration. Snails from Roselle, and snails from Bruna for comparison, were acclimated at 20°C for 5 days before the start of the experiment. This unnatural acclimation protocol, which is *de facto* a ‘common garden’ approach, allowed us to compare the response of the two groups across the same range of temperatures. Thirty specimens (shell length, 2.8–16.6 mm) of each population were placed in a circular plastic container (diameter, 15 cm; depth, 10 cm) and the water temperature was increased at a rate of 2°C^*^h^−1^ until the maximum temperature was reached. The snails were continuously monitored and at each temperature the number and the size of specimens that had migrated outside the water up the wall of the container and those that were inactive in water were recorded. Once a snail emerged from the water or withdrew inside its shell, it was removed from the experimental container. Each trial was replicated three times and a total of 224 snails were tested.

### Statistical analysis

The null hypothesis of no differences among respiration rates and righting times for different populations and temperatures was tested using a univariate permutational analysis of variance (PERMANOVA; Anderson, 2001).

To test the null hypothesis that the frequency of inactive (Bruna) or migrating (Roselle) specimens were not different across experimental temperatures, we performed PERMANOVA, taking into account that the measures were repeated on the same experimental specimens. To do this, the experimental design included the following factors: ‘temperature’ (number of levels were equal to the number of experimental temperatures in each trial) and ‘replicate’ (three levels; random and nested in ‘temperature’).

Similarity matrices were computed by using Euclidean distance on square-root-transformed data or arcsin of square root in cases of data expressed as percentages. All analyses were based on 999 restricted permutations under a reduced model of raw data and Type III sums of squares (Anderson *et al*., 2008). *Post hoc* pair-wise *t*-tests were used for multiple comparisons of means when PERMANOVA showed significant differences.

Linear regression was used to relate average shell length (mm) of snails becoming inactive in water (Bruna) or migrating outside the water (Roselle) with increasing experimental temperature. Statistical analyses were performed using PAST (Hammer *et al*., 2001) procedures and fitting of linear regressions were implemented with SigmaPlot v.11.

## Results

**Figure 2:**
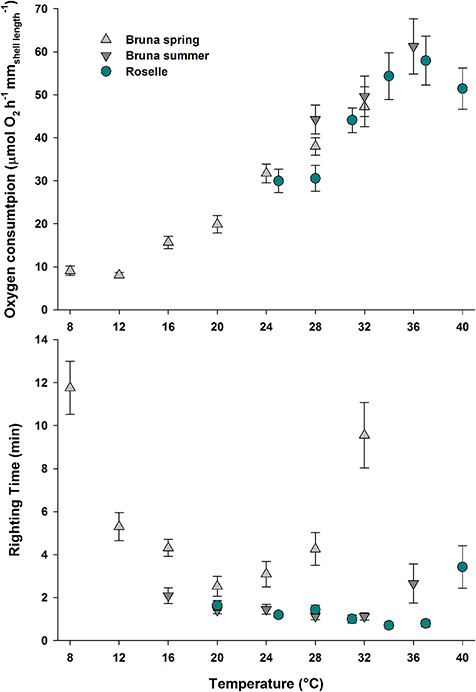
Oxygen consumption rates and righting performance along temperature ramps (2°C^*^h^−1^), exhibited by *M. etrusca* from a temperate seasonally fluctuating stream (Bruna) and constantly hot water (Roselle). The acclimatization temperatures of experimental specimens from Bruna were 20°C in spring and 27°C in summer, whereas in Roselle temperatures were consistently within the range of 35–36°C. (**A**) No sign of metabolic compensation emerged, following temporal (seasonal) or spatial (geographical) patterns of temperature variation, as oxygen consumption rates were consistent across different acclimatization temperatures. (**B**) The righting performance exhibited by the two populations, shown to be maximized at higher acclimation temperatures.

Oxygen consumption rates were consistently positively correlated with temperature (Fig. 2, Table 1). The rates of spring and summer cohorts of Bruna were recorded up to 32 and 36°C, respectively. These temperatures represent the thermal tolerance limits for activity of the populations naturally acclimated at 20 (spring) and 27°C (summer); beyond those limits, snails became inactive and withdrew inside their shells. Interestingly, no differences in oxygen consumption rates existed at 28 and 32°C between different seasons (Table 1). The animals from Roselle showed an increasing oxygen consumption rate until 37°C. At 40°C, respiration rates did not increase further (Table 1).

**Table 1:** Results of univariate PERMANOVAs carried out on the changes in *M. etrusca* oxygen consumption rate (MO_2_), righting time and migration frequency across experimental temperature ramps

Experiment	Sample	*n*	df	MS	*F*	Pair-wise
MO_2_	BSP	192	7	56 959	58 557 ^**^	8, 12 < 16, 20 < 24 ≤ 28, 32
	BSU vs BSP	73	1	3356	2292 ns	
	ROS	97	5	13 205	9179 ^**^	25, 28 < 31, 34 ≤ 37, 40
Righting	BSP	270	7	332 650	11 982 ^**^	20, 24 ≤ 12, 16, 28 ≤ 8, 32
	BSU	196	5	602	1769 ns	
	ROS	277	6	1738	4510 ^**^	37, 34, 31 ≤ 28, 25 ≤ 20, 40 20°C: BSU < BSP; BSU = ROS, ROS=BSP
	ROS-BSP-BSU	365	4	15 734	2404 ^*^	24–25°C: BSP_24_ < BSU_24_ = ROS_25_ 28°C: BSP < BSU = ROS
Migration (frequency)	BSP (inactive)	24	6	693	47 562 ^**^	32, 33, 34 < 35 < 36, 37 ≤ 38
	BSU (inactive)	27	7	1027	33 060 ^**^	33, 34, 35, 36 < 37, 38 < 39, 40
	ROS (migrated)	21	5	423	28 405 ^**^	36, 37 < 38 < 39, 40, 41

The experimental *n* (*n*), degrees of freedom (df), variance (MS), value of the pseudo-F ratio and probability levels are shown (^*^, *P* < 0.05; ^**^, *P* < 0.01; ns, not significant). In the last column, the results of pair-wise tests for experimental temperature comparisons are shown. Sample abbreviations are as follows: BSP, Bruna Spring; BSU, Bruna Summer; ROS, Roselle.

The snails from Bruna showed an optimal mobility performance at 20°C in spring, coincident with the acclimatization temperature, while performance tended to decline at both higher and lower temperatures (Fig. 2). The slowest righting times recorded were at 8 and 32°C, not differing from each other (Table 1). Mobility performance was enhanced in summer (acclimatization 27°C), particularly at high temperatures, with snails showing the fastest righting times between 28 and 32°C, although differences among experimental temperatures were not significant (Fig. 2, Table 1). At 36°C, the average righting time tended to increase (Fig. 2, Table 1). The comparison among righting times of two cohorts from Bruna at 16, 20, 24, 28 and 32°C revealed that the snails acclimatized to the summer temperature had a consistently faster mobility performance than those in spring (PERMANOVA, df = 4, F = 7.43, *P* < 0.01). The snails from Roselle performed optimally between 31 and 37°C, with a significant increase in righting time at 20 and 40°C (Fig. 2, Table 1). Between the two populations, the comparison of performances at 20, 24–25 and 28°C showed that animals from Roselle and from the summer cohort of Bruna performed better than those acclimatized to spring temperatures (Table 1).

**Figure 3:**
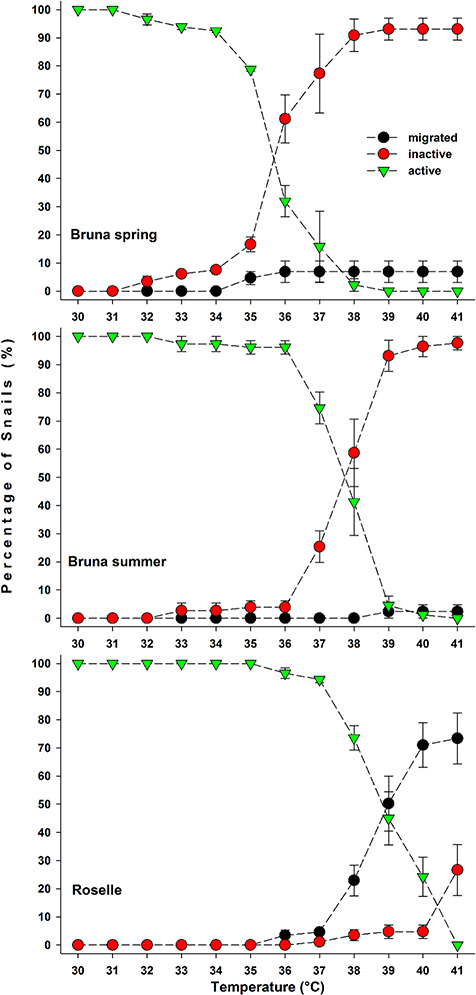
Behavioural response of the two populations of *M. etrusca* to gradual temperature rise (2°C^*^h^−1^). The population specialized to seasonal water temperature fluctuations (Bruna) showed a wider thermal window of tolerance in summer, when the acclimatization temperature was high (B, 27°C), as testified by the shift of the upper temperature threshold inducing cessation of activity. Only a very small proportion of specimens (6/164) migrated outside the water in response to hot water temperatures. Specimens acclimatized to the constant temperature of 36°C (Roselle) were naturally induced to migrate outside the water when experimental temperature reached the 38–39°C threshold.

The heat-induced migration outside the water was virtually absent in both cohorts from Bruna (Fig. 3, Table 1). In fact, snails tended to withdraw inside their shells remaining inactive in the water when the heat limit was reached. The onset of this behavioural response occurred at 36°C for the spring cohort, when 61.15 ± 8.57% of individuals were induced to aestivate in the water. This heat limit appeared to shift at 38°C for the summer cohort, with 58.70 ± 11.92% of individuals recorded as inactive in water. At 39°C 2.38 ± 2.38% of snails migrated out of the water whereas 4.55 ± 3.20% were still active in the water. At 40°C the percentage of active snails further decreased to 1.19 ± 1.19%. At 41°C no active snails were observed in the water. The snails from Roselle showed a markedly different pattern of thermally induced migration outside of the water and the onset of this behaviour occurred at 38°C (Fig. 3, Table 1). At 40°C, 70.98 ± 7.93% of snails migrated outside the water. At 41°C, the last snails remaining in the water (26.67 ± 9.09%) did not migrate but instead withdrew inside their shells (Fig. 3, Table 1).

**Figure 4:**
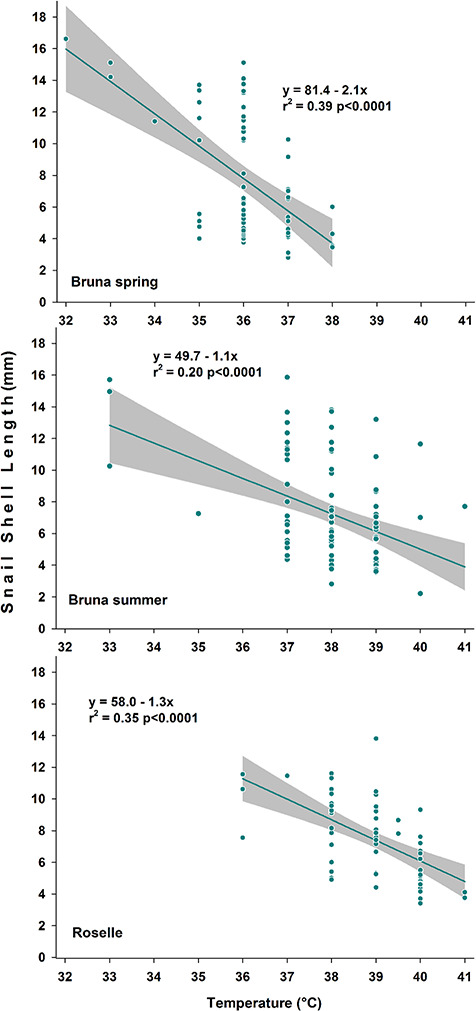
Linear regression between the average shell length of experimental snails becoming inactive in water or migrating outside the water (**A**, Bruna spring; **B**, Bruna summer; **C**, Roselle) and the experimental temperatures. In both populations, larger specimens were more sensitive to higher temperature, as demonstrated by the smaller average size of snails observed active in the water across the experimental temperature increase (2°C^*^h^−1^).

The susceptibility to increasing temperature was demonstrated to be size dependent in both populations, as shown by the negative relationship between shell length and temperature (Fig. 4; Bruna spring: *F* = 38.89, *n* = 64, *P* < 0.001; Bruna summer: *F* = 23.30, *n* = 97, *P* < 0.001; Roselle: *F* = 31.97, *n* = 63, *P* < 0.001). Specifically, large animals (shell length > 10 mm) from Bruna became inactive and those from Roselle emerged from the water at lower temperatures compared to snails of medium/small size (shell length < 10 mm; Fig. 4).

## Discussion

### Thermal sensitivity of performance

The capacity to sustain aerobic metabolism during acute warming represents a pivotal mechanism regulating the breadth of their thermal niche and the limits of heat-dependent performances in many aquatic ectotherms (Pörtner, 2010; Clark *et al.*, 2013; MacMillan, 2019). The exponential rise of oxygen consumption ideally describes the thermal response to abrupt variations in the vast majority of water breathing organisms (Gillooly *et al*., 2001; Brown *et al.*, 2004; Giomi and Pörtner, 2013). Several aquatic ectotherms shift their aerobic performance to match their habitat temperature range (e.g. Sokolova and Pörtner, 2003; Wittmann *et al*., 2008; and recently conceptualized in Sinclair *et al*., 2016). These shifts occur over short time scales when ectotherms acclimatize to seasonal temperature fluctuations and across multiple generations when species evolve into new thermal niches.

Surprisingly*, M. etrusca* showed non-acclimation of temperature-related metabolism, since the temperature-dependent oxygen consumption rate was consistent, regardless of seasonality and habitat features. Specifically, the oxygen consumption rate of active individuals is not fitted to the thermal regimes of the specific locations nor to compensate for the seasonal shift of the thermal niche. The metabolic rates of the three cohorts differed exclusively for the maximum temperature at which they were sustained. The spring and the summer cohorts of the Bruna population showed a sizable increment of the thermal limit for oxygen consumption from 32 to 36°C, indicating that certain acclimatization may partially compensate for the seasonal expansion of the thermal niche at higher temperatures. Beyond these thermal limits, it was not possible to take additional measurements since the animals withdrew inside their shells. This response to thermal extremes is similar to the behaviour that the Bruna population adopts in winter, when the thermal regime shrinks to low temperatures and *M. etrusca* become inactive on the stream bed (Bartolini and Giomi, pers. obs.). On the contrary, the snails from Roselle did not withdraw inside their shells but they were able to sustain the increase of metabolic rate up to 38°C, while it progressively declined when animals were subjected to further warming. This physiological response to warming, distinctive of the population in Roselle, is similar to the trends shown by the majority of aquatic ectotherms and suggests a progressive switch to anaerobic metabolism when the capacity to extract oxygen from the water becomes compromised (Pörtner, 2010; Giomi and Pörtner, 2013). The shift to anaerobic metabolism has been demonstrated in intertidal gastropods, which showed to be able to survive at temperature >4°C beyond the limit of Arrhenius break temperature (Han *et al.*, 2017). Interestingly, in contrast to the population of Bruna, the Roselle population rarely becomes inactive in the stream but instead regularly crawls outside of the hot water onto the banks (Bartolini and Giomi, pers. obs.).

The comparison of the righting time, as a proxy for mobility, between the spring and summer cohort of the Bruna population reveals a seasonal shift and widening of the thermal performance breadth. In fact, the summer cohort showed greatest activity at higher temperatures, maximizing their mobility performance more efficiently than the spring cohort. Interestingly, this trend also persists at low temperatures, representing the acclimatization condition of the spring cohort (around 20°C), which showed consistently slow righting times. This observation is in agreement with the seasonal pattern of activity exhibited by the Bruna *M. etrusca* population. During winter months, snails undergo a suspension of somatic growth that resumes in spring (Bartolini *et al.*, 2017).

Even though the Roselle population showed a thermal window of activity larger than the summer cohort of Bruna, with an upper limit over 37°C, the righting time of the two groups overlapped for most of the temperatures. This result reveals the capability of the population that inhabits the seasonally variable environment to match the performance attained by the thermal specialist population. In this respect, the peak of the Roselle snails’ mobility performance matches the constant high temperatures of this stream, which is fed by a hot thermal spring (annual range, 35–38°C). Interestingly, the thermal optimum for mobility, represented by the fastest righting time, differs slightly from the critical temperature, suggesting that this population optimized its performance in the narrowest range close to the upper tolerance limit.

These findings provide an interesting example of the phenotypic plasticity of gastropods to heat tolerance and underline the importance to downscale physiological investigations to the most relevant local level. In fact, although metabolic rates of typical ectotherms increase with temperature, some species are able to restrain heat sensitivity through metabolic depression (Houlihan and Innes, 1982; Sokolova and Pörtner, 2001; Marshall and McQuaid, 2011; Marshall, *et al*., 2011; Verberk *et al*., 2016). For example, intertidal gastropods have the capacity to adopt a temperature-insensitive metabolism, even below their limits of heat tolerance (Garrity, 1984; Marshall *et al*., 2011). Snails can rest inside their shell, lowering their metabolic rate and minimizing the energy loss that otherwise would be imposed by cyclical stressful conditions, such as daily peak temperatures. In the present study, we found evidence of both physiological mechanisms in geographically separated populations of the same species. Specifically, the snails in Roselle maximize their metabolism and performances close to the upper end of thermal tolerance, displaying a progressive decline when the heat limit is exceeded. Conversely, the snails in Bruna, active over a wider thermal window, respond to adverse temperature extremes, both at the low and high end of the niche, by retreating within their shells showing a good capacity to down-regulate cellular metabolic demand.

Accordingly, we hypothesize that population-specific behaviours have promoted the divergence of the thermal physiology of *M. etrusca* at an extremely local scale.

### Thermoregulatory behaviour

**Figure 5:**
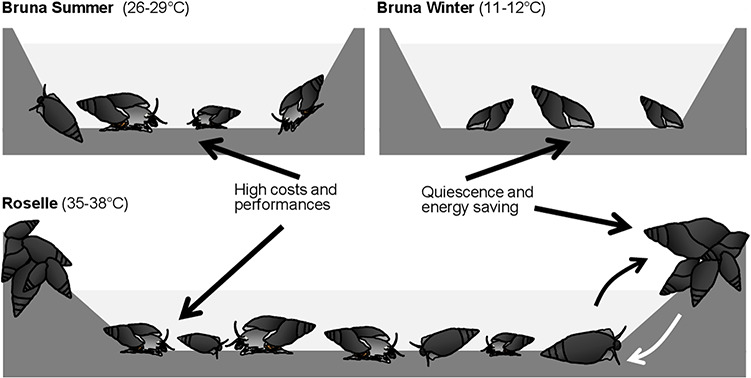
Conceptual model showing the pattern of *M. etrusca* activity, reflecting the specialization in streams with different thermal regimes. In the Bruna stream, temperature ranges between 12 and 27°C according with a seasonal fluctuation: in winter, when the water temperature (Tenv) decreases below the optimal temperature for activity (Topt), *M. etrusca* suspends somatic growth and reduces the activity in water, wintering on the stream bed. With the seasonal rise of water temperature snails resume both activity and somatic growth. In the Roselle stream, snails experience constant temperature conditions of 35–38°C coincident with the optimal temperature for metabolic and activity performances. However, to avoid continuous exposure to such a hot microclimate, snails emerge from the water on the stream banks, aestivating for periods in a milder thermal niche (Tpref).

For the population of Roselle, we demonstrated that 38°C represents the temperature threshold inducing the onset of migration outside the water and aestivation phase, when snails are observed withdrawn inside the shell. This heat limit corresponds to the maximum temperature recorded in this stream (Bartolini *et al*., 2017). During several periods of field observation at Roselle, we documented solely the occurrence of animals active in hot water or laying inactive on the banks a few centimetres above the water level. To explain these observations, we hypothesized that periodical emersion behaviour would be the strategy adopted to minimize the costs associated with settlement in this extremely hot site (Fig. 5). The microhabitat occupied by the snails along the stream banks is indeed characterized by air temperatures that are generally lower than aquatic temperatures (Bartolini and Giomi, in prep). Conversely, within the stream, the marked temperature dependence of metabolic rate implies a high cost of maintenance diverting energy expenditure away somatic growth and reproductive investment (Brown *et al*., 2004; Nisbet *et al*., 2012). Thus, *M. etrusca* in Roselle commutes between two dissociated thermal niches to recurrently aestivate in the cooler environment. While resting on the river banks, the snails of Roselle are able to optimize their energy budget by means of a good capacity to down-regulate cellular metabolic demand. This behaviour likely maximizes the snail individual fitness. Similarly, the active selection between habitats characterized by disjointed thermal regimes has been documented in several ectotherms. A separation between thermal niches, where the optimal temperature for performance differs from the preferred temperature that minimizes energy expenditure, has been observed in situations of limited resources, strenuous habitat conditions or under growth and reproductive constraints (Huey, 1991; Angilletta 2009). The sockeye salmon (*Oncorhynchus nerka*) performs daily migrations to deep cold water under conditions of food limitation to reduce metabolic costs, even though the optimal temperature for foraging is in the shallow warmer habitat (Bevelhimer and Adams, 1993). The behavioural strategy of the population in Roselle would allow for highly efficient foraging in hot water where the mobility performance is optimal and the maximization of growth when aestivating in a low energy demanding environment (Fig. 5).

The shell of gastropods allows several species to colonize unexploited habitats, constituting a physical barrier to harsh temperatures and adverse conditions and providing a retreat to endure physical stress and avoid predation. Aestivation is a well-known strategy showed by freshwater and terrestrial snails, which are able to avoid adverse environmental conditions, such as periodic aridity or deterioration of physico-chemical water conditions. Aestivation is often accompanied by some degree of ‘escape’ from unsuitable environment, including horizontal or vertical migration. Horizontal migrations allow snails to actively disperse in search for more favorable areas (Poznańska *et al.*, 2015; Jokinen, 1978). Vertical migrations include burial into wet sediment (Ferreira *et al.*, 2003) or crawling outside water (Proum *et al.*, 2017).

Intertidal marine snails are able to thermoregulate or to suspend their activity during low tide and become inactive during periods of emersion (Garrity, 1984; Munoz *et al*., 2005; Marshall and McQuaid, 2011; Marshall, *et al*., 2011; Verberk *et al*., 2016). Several freshwater snails adopt aestivation as a strategy to survive desiccation caused by periodic drought, showing astonishingly capacities to survive in the absence of water. Pulmonate snails are able to withstand several months of environmental drought (Jokinen, 1978), with extreme examples in tropical aestivating species (e.g. Richards, 1967). Gill-breathers can tolerate shorter periods of desiccation ranging from few days to several weeks (e.g. Poznańska *et al.*, 2015). Prolonged aestivation frequently involves transition into a hypometabolic state and anaerobic metabolism (Storey and Storey, 2012; Ferreira *et al.*, 2003). On a short time scale, *M. etrusca* appears to periodically aestivate, purportedly to avoid the prolonged exposure to high water temperatures. We suggest that during this phase, *M. etrusca* undergoes to short-term metabolic adjustment (likely hypometabolism as described by Marshall and McQuaid, 2020). As a matter of fact, individuals are observed active in the water across every season (Bartolini *et al.*, 2017). In other words, Roselle population shows an adaptive behavioural emergence from water, as opposed to emergence from the water in response to respiratory stress, as is observed in many aquatic animals when water PO_2_ declines.

We observed that most snails from Bruna became inactive in water and withdrew inside their shells when exposed to extreme temperatures; however, a small number of individuals migrated outside the water (overall 6 specimens out of 164 tested). This suggests that this behavioural trait remains inherent of the species and has promoted the intra-specific specialization to extreme thermal conditions by means of highly selective exaptation (Gould and Vrba, 1982). The fact that our experimental evidences come from a common-garden approach (snails acclimated at 20°C for 5 days), corroborates the hypothesis that an adaptive behaviour to specific microclimate conditions has been selected in the Roselle population. Through downscaling our study to the population level, we recognized that the divergence of behavioural features clearly distinguishes the animals of Bruna and Roselle providing *M. etrusca* with distinct mechanisms to colonize an array of sites characterized by highly divergent thermal regimes. It is worth mentioning that body size is revealed to trigger these behavioural traits, being inversely related to the thermal tolerance. Indeed, both the emersion and the withdrawn behaviours were elicited at lower temperature in larger snails. Interestingly, another population of *M. etrusca* that lives in a constantly warm stream (30–35°C; Venturina) does not exhibit clear emergence behaviour. In this site, snails attain high growth rates but have a short life span resulting in a population dominated by small specimens where a clear structure in size classes is absent (Bartolini *et al*., 2017). Conversely, populations from Bruna and Roselle appear well structured due to different strategies (quiescence and migratory behaviour, respectively), with a high frequency of large specimens (Bartolini *et al*., 2017). In other words, if a successful behavioural specialization is not fixed in a certain population when restrained to a hot spring, this results in limited demographic structure.

### Conservation perspectives

In recent years, the knowledge of species physiology (Wikelski and Cooke, 2006; Cooke *et al*., 2013, Cooke *et al*., 2014) and behaviour (Anthony and Blumstein, 2000; Berger-Tal *et al*., 2011) has been integrated into conservation science to help discover practical solutions to counteract the loss of biodiversity. Within this framework, we downscaled the characterization of physiological and behavioural traits to populations specialized to distinct microclimatic niches. The study of *M. etrusca* thermal physiology highlights the need to focus on local microclimatic niches, especially when intraspecific specialization occur at a fine geographic scale. Similarly, conservation actions should take into account the occurrence of specific traits that enable populations to cope with local variability, particularly in extremely selective environments. We believe that our findings have specific practical implications for the conservation of *M. etrusca* and generally support the need to downscale conservation efforts to the most relevant evolutionary and ecological level*.*

In the future, introduction/reintroduction programmes could represent a strategy for restocking *M. etrusca* both in streams where local populations become extinct, once the causes of extinction have been mitigated or removed, and in new streams (IUCN, 2013). However, populations from temperate streams with seasonal variation of the thermal regime may lack of physiological and behavioural traits enabling the colonization of hot streams. Therefore, beside genetic consideration concerning the most appropriate population suited for a potential introduction (Neiber *et al*., 2020), it would be necessary to select the most appropriate stock according to rigorous physiological and behavioural criteria.

*Melanopsis etrusca* populations appear to be currently isolated by geographical barriers. Recent genetic evidence is consistent with a pattern of isolation by distance and shows a differentiation into a western, a central (including Bruna) and an eastern (including Roselle) group of populations (Neiber *et al*., 2020). In this scenario, an assessment of possible detrimental genetic processes (e.g. genetic loads) affecting isolated populations or a group of populations would be required (Martin *et al*., 2012). This information, combined with the evidence of adaptive innovations, would pinpoint the necessity of specifically designed conservation actions. In fact, our study suggests that the Roselle and Bruna populations are not ecologically exchangeable (Crandall *et al.*, 2000) and thus could be considered partial Evolutionary Significant Units (de Guia and Saitoh, 2007).
